# The Book World of Medicine and Science

**Published:** 1893-11-18

**Authors:** 


### The Book World of Medicine and Science.

Evolution and Ethics. The Romanes Lecture, 1893. By Thomas H. Huxley, F.R.S. (Macmillan and Co.)

The importance of this brief contribution to the ethical problems of the day can hardly be over-estimated. It is practically a gathering up, by the man best qualified to assume the task, of the results so far as they concern human conduct of the gigantic scientific achievements of the century. To what extent have the new truths modified old ideals? How have they affected the varying human standard of right and wrong? Does the doctrine of evolution tend to elbow from its throne the doctrine of self-sacrifice, and set up in its stead a monstrous image of inevitable selfishness? These questions, rashly and too conclusively argued in the pulpit, need before all things to be steeped in a dry light of impartiality, and it will readily be admitted that Mr. Huxley's light (differing widely in that respect from his style) is about as dry for the purpose as any to which access can be had. Nothing can be clearer than the lecturer's repudiation of the theory often attributed to evolutionists, of moral fatalism, excluding human responsibility, on the ground of obedience to that law which results in the "survival of the fittest." The "fittest," he points out, depends purely on conditions, and is by no means necessarily the "best" ("if our hemisphere were to cool again, the survival of the fittest might bring about in the vegetable kingdom a population of more and more stunted and humbler and humbler organisms"); but social progress, opposing at every step the cosmic process, aims not at "the survival of those who may happen to be the fittest in respect of the whole of the conditions which exist, but of those who are ethically the best." The ethical progress of society depends, in fact, "not on imitating the cosmic process, still less in running away from it, but in combating it." Man is to be set to subdue nature to his highest ends, and the great work which science has wrought may be summed up "in the solid foundation we have acquired for the hope that such an enterprise may meet with a certain measure of success." Substituting, then, for the old formula of "the world, the flesh, and the devil," the evolutionist's "cosmic process," the new code of ethics is found to differ not so greatly from the old. "In place of ruthless self-assertion, it demands self-restraint; in place of thrusting aside or treading down all competitors, it requires that the individual shall not merely respect, but shall help his fellows; its influence is directed not so much to the survival of the fittest as to the fitting of as many as possible to survive." And for ever man must "cast aside the notion that the escape from pain and sorrow is the proper object of life."

The Diseases of Childhood (Medical). By H. Bryan Donkin. (Charles Griffin and Co.)

Of bookmaking there is no end. And as one travail after another is brought to the birth, the eye of the weary reader for the most part scans the new arrival somewhat apathetically, the chances seeming to be all against anything original, or, even in many cases, likely to give pleasure in the reading.

But this was not the frame of mind with which this book came into our hands. Already familiar with Dr. Donkin as a writer who, when he has written, for he has hitherto been somewhat jealous of his pen, has always gone straight to the point; who has always moved well within the circle of his

**THE BOOK WORLD OF MEDICINE AND SCIENCE**—*continued.*

own knowledge, or at any rate of his experience, for few but the ignorant attain to certainties in medicine; who always looks at things from his own point of view, and in consequence always writes what is worth reading and worth thought, one felt inclined to exclaim, not having heard that there was an expectation in this direction, with many apologies to our author for so tawdry a clothing to the idea,

It comes as a blessing to medical men,
This book from the Shadwell and Westminster pen.

It did, indeed, come to us as a pleasurable surprise, and now on finishing its perusal we are in no way disappointed.

It consists, as the author tells in his preface, of the records and recollections of nearly twenty years experience at the East London Hospital for Children, and includes the substance of some lectures given at the Westminster Hospital, and of contributions to the Westminster Hospital Reports. It would seem indeed that these lectures had determined the scope and method of Dr. Donkin's teaching, for his book is essentially a clinical manual, and may be said to be composed throughout of short clinical lectures which to our mind most compare with Henoch's "Lectures."

We shall not attempt to review the book in detail. We have read most of it carefully. There are naturally parts where the author's judgment and observation have led him to conclusions other than those which the reader might be inclined to support; but there is never a place where the facts have not been stated with the most scrupulous regard to the teachings of experience, and there are several places —the question of the undue tolerance of belladonna in childhood is one such that occurs to one at the moment —where his teaching is in opposition to the rather widely prevalent one, but with which a mature experience is very much inclined to agree.

Take it all round the book is one of the most uniformly pithy that we have read for a long time, and it may be added that the pith is invariably succulent. It is a book that everyone should read, and all who do this will certainly often return to it.

